# Assessment of Environmental Distribution of Lead in Some Municipalities of South-Eastern Nigeria

**DOI:** 10.3390/ijerph7062501

**Published:** 2010-06-10

**Authors:** John Kanayochukwu Nduka, Orish Ebere Orisakwe

**Affiliations:** 1 Environmental Chemistry and Toxicology Research Unit, Pure and Industrial Chemistry Department, Nnamdi Azikiwe University, P.M.B. 5025, Awka Anambra State, Nigeria; E-Mail: johnnduka2000@yahoo.co.uk; 2 Toxicology Unit, Department of Clinical Pharmacy, Faculty of Pharmacy, University of Port Harcourt, Rivers State, Nigeria

**Keywords:** lead, roadside surface soil, rain water, dust particles, municipal cities, Nigeria

## Abstract

Lead (Pb) levels were measured in roadside surface soils, dust particles and rain water samples from the urban cities of Enugu, Awka, Onitsha, Nnewi, Aba, Port Harcourt and Warri in Southern Nigeria in 2007 and 2008. Samples were collected during the dry season, while rain water samples were collected during the early rain (April–June), mid rain (July–August) and late rain seasons (September–October) for the two years. Soil samples were collected from traffic congested roads, dust was collected by tying a plastic basin on a pole 1.5 m above ground level and leaving it for 45 days. Rain samples were collected from three equidistant points. Samples were analyzed by AAS. The highest soil Pb of 120.00 ± 0.00 and 80.36 ± 0.00 mg/kg were reported in Onitsha for 2007 and 2008, respectively. Nnewi showed 33.40 ± 0.01 and 4,238.29 ± 0.00 mg/kg for 2007 and 2008. Aba had 22.56 ± 0.01 and 21.28 ± 0.00 mg/kg for 2007 and 2008. Higher concentrations were recorded for Nnewi and Port Harcourt in 2008 than in 2007. Enugu had more in 2007 while Awka had more in 2008. Dust Pb ranged from 0.13–0.49 mg/kg and 0.15–0.47 mg/kg for 2007 and 2008, respectively. Rain samples had the least Pb concentration, ranging from 0.103 ± 0.000 to 0.163 ± 0.046 mg/L. We may conclude that Nigerians are exposed to environmental Pb.

## 1. Introduction

Lead is a metal which has been associated with human activities for several decades. It is a common industrial metal that has become widespread in air, water, soil, and food. In ancient civilizations, the uses of lead included the manufacture of kitchen utensils, trays, jugs and other decorative articles that are found in home. A consequence of its many industrial application had led to its wide distribution in the environment today. A compilation has been made of 120 occupations, e.g., auto-mechanic, painting, printing, welding, *etc*., that may involve exposure to lead [1]. It has been established that all humans have lead in their bodies, primarily as a result of exposure to man-made sources [2]. Before now organic lead compounds (tetraethyl lead and tetramethyl lead) were extensively used as additives in gasoline but at present unleaded gasoline is used in most countries of the World. Africa has contributed substantially to global lead pollution [3–6]. Nigeria is not an exception. In Nigeria, gasoline with an average Pb content of 0.66 g/L remains in use [7]. With high automobile importation, the national consumption of petrol in the country is estimated at 20 million litres per day, with about 150 people/car/city, therefore close to 15,000 kg of lead is emitted into the environment through combustion [8]. The environmental routes of lead exposure in Nigeria are as varied as its toxicity to humans. We have established high lead levels in soil at refuse dumpsites as a result of decomposition and deterioration of wastes [9]. A high volume of fairly used electronics such as computers and their accessories, TV sets, fax machines, cell phones, *etc.*, importation, and experts estimate that 25% to 75% of these material are irreparable junk, and because the country lacks the capacity to safely deal with electronic waste, they wind up in landfills and informal dumps where they decompose and release toxic metals such as lead, cadmium and mercury into the environment [10]. We recently reported high levels of lead from flaking paint chips from buildings in Southeastern Nigeria [11]. Increased globalization and outsourcing of manufacturing has increased the likelihood that painted products with unacceptably high levels of lead are being traded between China and Africa. Takaoka *et al*. [12] have shown that both gasoline lead of the past and paint chips contributed to increased lead concentration in the surface soil of playgrounds in Tokyo, though the contribution of paint chips is smaller than that of gasoline lead. A study in Nigeria has suggested that automobile emissions are a major source of lead exposure as the highest concentration of lead and zinc in regions studied were recorded in the commercial areas of Abuja [13]. Preliminary studies showed that lead air emissions at ground level in Lagos (Nigeria) are far higher than levels in cities like London and New York, but similar to those of Brazil or the Caribbean, due to perturbation by roadside traffic [7]. The severity of the problem in the study area results from the poor road network and the dilapidated nature of the existing roads, coupled with large volume importation of used vehicles leading to high number of irreparable and decomposing automobiles littered on the roads of Southeastern Nigeria, hence more road traffic congestion, and this twin problem can release lead (Pb) in the environment. Children run the greatest risk of lead poisoning in inner city areas where inhalation and ingestion of metals like lead and cadmium from dust and ashes are common [7]. Hyperactivity, anorexia, decreased play activity, low intelligence quotient, and poor school performance have been observed in children with high lead levels [14]. Lead crosses the placenta during pregnancy and has been associated with intrauterine death, prematurity and low birth weight. Adult exposure occurs more frequently in the workplace and primarily involves the central nervous system. Symptoms of hemoepoitic system involvement include microcytic, hypochromic anemia with basophilic stippling of the erythrocytes [15]. Although the Nigerian government has not paid serious attention to environmental pollution and degradation, the aim of this work is to support the overwhelming evidence that lead pollution is on the rise in Nigeria and the exposure routes are as varied as its toxicity [9,10,13,16–20], while appreciable reduction has been achieved in the developed countries of the world [21]. Though lead pollution can be controlled by regulating the importation of electronics and automobiles, legislation, stoppage of use of lead in paint and other industrial manufacture, strict monitoring and control, no serious attempt has been made in any of these areas in Nigeria. It is advocated that any legislation to check lead exposure to humans should be based on genuine scientific evaluation of the available data [22].

The present study has assessed the environmental phase distribution of lead in some municipal cities of Southeastern Nigeria. It is believed that these data will assist policy makers and public health providers in Nigeria in knowing the size of the problem of environmental pollution in Southeastern Nigeria, since the region is the major hub of road transport and industrialization.

## 2. Materials and Method

**Figure 1.**
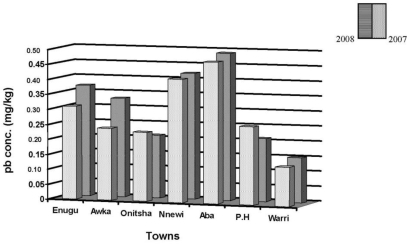
The concentration of lead in mg/kg of dust collected in the studied towns during 2007/2008.

The level of lead (Pb) was examined in roadside surface soils, rainwater samples and harmattan dusts of seven major cities in South Eastern Nigeria, namely Enugu, Awka, Onitsha, Nnewi, Aba, Port-Harcourt and Warri (Figure 1).

In each city, soil samples were collected during the dry season of 2007 and 2008 from two roads designated Road 1 and Road 2 and generally known to have high vehicular movement. On each road two points labeled Point I and Point 2 and approximately 500 m apart were marked. At each point, three surface soil samples, 10 g each, were collected in a triangular array from three points approximately 10 m apart from each other, and soil control samples were collected approximately 120 m away from the tarred road. All were stored in black polythene bags before digestion and analysis. The soil samples are fine to medium sand in texture. They were ground, mixed and divided into fine particles that could pass through a 0.5 mm sieve. Digestion was done by placing 2 g of soil in a conical flask, adding 15 mL of concentrated nitric acid and perchloric acid at a ratio 1:1 and heating in a fume cupboard at a temperature of 105 °C until the volume was reduced to slightly less than 1 mL, then the sample was allowed to cool, 10 mL of de-ionised water were added, and the solution stirred and filtered and made up in standard volumetric flask. Harmattan dust samples were collected by tying a small plastic container at 1.5 m above the ground level at a particular pole on each road in each city and allowing it to stand for upwards of 45 days. The dust samples were sieved and 1 g was digested as described above. The results were the average of three measurements per road.

Rainwater samples were sampled three times each year between April–October 2007 and 2008, namely April–June, July–August and September–October, to represent early rain, mid rain and late rain of each year (2007 and 2008). In each city, the samples were collected from three locations equidistant from each other using a clean plastic basin fastened to a table 1.5 m above ground level and 75 m away from tall buildings and trees, the water samples were filtered, 10 mL of conc. nitric acid was added to 5 mL of rainwater samples and the mixtures allowed to stand for 135 min until they became colorless. Lead levels of soil, dust and rainwater sample filtrates were determined at a wavelength of 217.0 nm using atomic absorption spectrophotometry as described by Piper [23]. The results were presented as mean ±SEM. Soil cation exchange capacity were determined as described by Brady and Weil [24]. Organic content was determined as described by Emedo *et al*. [25]. Soil pH was determined by weighing 20 g of air-dried soil (passing through a 0.5 mm sieve) into a 50-mL beaker, then adding 20 mL of distilled water, allowing to stand for 30 min, while stirring occasionally with a glass rod. A pH meter electrode was inserted into the partly settled suspension and the pH measured; the result was reported as “soil pH in water”. Rain water pH was determined after filtration by standardizing with 0.1 M KCl and the electrode of the pH meter was immersed into the sample in a flask. The instrument turned on and the pH was read directly. The limit of detection for Pb was 0.01 ppm respectively with blank values reading as 0.00 ppm in deionized water with electrical conductivity value of lower than 5 μS/cm. Samples were analyzed in triplicate.

## 3. Results

Appreciable quantities of lead levels were determined in all the samples, with soil samples having the highest values. Onitsha, Aba, Nnewi and Port Harcourt soils had the highest lead content for both years. Highest values of 120.00 ± 0.00 mg/kg and 80.36 ± 0.00 mg/kg of lead were reported in Onitsha for 2007 and 2008. Nnewi (33.40 ± 0.01 and 4,238.29 ± 0.00 smg/kg) for 2007 and 2008. Aba (22.56 ± 0.01 and 21.28 ± 0.00 smg/kg) for 2007 and 2008. Higher concentrations were recorded for Nnewi and Port Harcourt in 2008 than in 2007, while Enugu had more in 2008 than in 2007, though the roads vary. The lead levels for each point (point of collection) on each road of all the cities vary, and the levels of Warri were smaller, followed by Awka and Port-Harcourt, respectively, in 2007, while in 2008, the levels of Warri were low and followed by those from Enugu (Tables 1 and 2).

The dust particle concentrations of lead (Pb) are in the range of 0.13–0.49 mg/kg and 0.15–0.47 mg/kg for both 2007 and 2008 for all the cities. More lead concentrations were found in dust particles of Warri, Aba, Nnewi, Awka and Enugu in 2008, while dust particles from Port-Harcourt and Onitsha had more lead in 2007 (Figure 1). Rain water samples had the least value of the metal when compared to those of surface soils and dust particles. Lead was not detected in the rain samples of Enugu and Awka, and mid rain samples of Onitsha and Nnewi for both years (2007 and 2008) under study.

Higher lead levels were recorded in the early and late rains in all the cities for both years. This may be because during early and late rain seasons, the rain is scanty and so may tend to contain more lead metal, while in the mid rain season there is heavy precipitation and so dilution may be an important factor (Tables 3 and 4). Cation exchange capacity and the pH of the soils are shown in Tables 5 and 6 while Table 7 depicts traffic volume. Due to insignificant amount of organic matter of the soil, the corresponding Table is not shown.

## 4. Discussion

The primary aim of this study was to determine the levels of lead (Pb) in surface soils, dusts and rain water samples to which individuals in selected municipal cities in Southeastern Nigeria are exposed. The results seem to be in conformity with other studies that environmental contamination can be caused by urbanization and development. Contamination of the environment includes that arising from agricultural activities, gaseous deposits from the air, waste water, sewage and industrial effluents. Industrial effluents may contain contaminants such as metallic ions that pose a threat to the natural ecosystem [26]. Metals are unique environmental and industrial pollutants in that they are found naturally distributed in all the phases of the environment [27]. Through a series of chemical and biochemical processes metals are concentrated and transformed into various products resulting in the amount of the metals being higher than their natural environmental concentration. Lead (Pb) stands out as the most ubiquitous metal in Nigerian environment, caused by automobile emissions [13], industrial effluents [28], paint flakes [11], refuse dumps [9] and electronic wastes [10]. Despite global awareness of the hazardous nature of lead, enforcement of legislation to mitigate lead pollution in Nigeria has not yielded many results. The values of our results, especially that of road surface soil is astonishing. Roads in Enugu has higher lead content in 2007 than 2008, while roads in Nnewi showed higher levels in 2008 than 2007. Aba roads had almost the same levels for both years under study. Onitsha showed higher lead levels in 2007 than 2008, with the highest level of 120.00 ± 0.00 mg/kg and Nnewi with 33.40 ± 0.01 mg/kg in 2007. Nnewi had the highest value of 4,238.29 ± 0.00 mg/kg while Port Harcourt had 89.75 ± 0.03 mg/kg in 2008 (Tables 1 and 2). For 2007, the total lead (Pb) level in Enugu soil from Road 1 is less than that from Road 2 (Point 1 plus Point 2), the same with Awka, Aba and Port –Harcourt, only Nnewi and Onitsha soil samples had more lead level in Road 1, while that of Warri for the two roads seemed equal. But in 2008, total soil lead (Pb) levels for Road 2 were less than that of Road 1 in Enugu and Awka, but more in the rest of the cities. The control values on each point for any road in all the cities are insignificant when compared with results (Tables 1 and 2). The total lead level for the two roads in each city shows that Enugu, Aba and Onitsha had more lead in 2007 while Awka, Nnewi and Port-Harcourt has more in 2008, and the Warri values were nearly equal for both years. Lead levels in each city seem to correlate with the level of development and economic viability. Nnewi, Aba, Enugu, Onitsha and Port Harcourt, with high volume of small and medium scale manufacturing concerns, high vehicular traffic, trade and being older in terms of governmental presence showed higher lead contents for both years. The exceptional high value of roadside surface soil in Nnewi at Point 2 (Road 2) in 2008 could be because the sample was taken at a roadside refuse dumpsite and the result agrees with our previous report [11].

Artisans such as car washes, panel beating, auto-mechanics, filling stations that use petroleum products and others like welding, painting, hair dressing, furniture making *etc*. are common features in the study areas, and may add to the environmental burden of lead on the roadside surface soils. The automobile spare parts market where sales and repair of vehicle spare parts are carried out release considerable amount lead to the environment [16]. Automobile exhaust is believed to account for more than 80% of air pollution in some urban centres in Nigeria. The levels of lead in Nigeria’s super grade gasoline is 600–800 mg/L [21,29,30] which may be higher than that of most developed countries. The high levels recorded in dust particles may impact on the inhalable particulate matter amongst the people especially commercial motorcycle transporters popularly known as “Okada”.

Respiratory abnormalities associated with occupational exposure to particulate insults in “Okada” (motorcycle) operators in Nigeria was studied by Orisakwe and co-workers and revealed serious health implications [31].

The highest level of lead (Pb) has been reported to occur in Nigerian Aviation gas (915 μg/mL) as compared to 200 μg/mL and 500 μg/mL in the US and Britain (UK), respectively [21].

Cation exchange capacity (the sum total of the exchangeable cations that a soil can absorb) is a function of a variety of soils and soil material; humus soil (with high organic matter) has high cation exchange capacity (CEC), [24]. Since our study samples are loose roadside sandy soils with very insignificant or no organic content (though the Table is not shown), the cation exchange capacity is therefore small (Table 5). The cation exchange of most soils increases with pH, at very low pH values, the cation exchange capacity (CEC) is also generally low; as the pH is raised (alkalinity), CECs increases. To obtain a measure of this maximum retentive capacity, the CEC is commonly determined at pH 7 or above, therefore the less the pH, of the soil, the less the cation exchange capacity (Tables 5 and 6). There is no direct relation between lead concentration and CEC, and this can be attributed to soil nature, absence of organic matter and flooding, as the cities are within the tropical rain forest zone of Nigeria [32]. The pH of rain water samples is in the range of 4.70–7.50 in all the cities (Table not shown), this agrees with our previous report in Awka, Warri and Port-Harcourt [11]. Though minor variation exists, the concentration of lead of roadside surface soil increases with increase in traffic congestion (Tables 1, 2 and 7) for both years under study. Several small, medium and large scale industrial manufacturing outfits such as electrical cable, battery, plastics, textiles, paper, brewery, vegetable oil, automobile parts manufacturing and assembling; in addition to fertilizer and tyre manufacturing, rubber and timber processing, iron ore, tin smelting and rolling mills as well as crude oil exploration, exploitation and refining that occur in some of the cities of study, can increase the concentration of lead in those cities, and previous reports support this view [28,33,34].

The lead levels for the soils, dust and rain water samples show a decrease in concentration from soil to dust, with rain water samples having the least, in all the cities for the two years under study. Though the lead (Pb) values of rain water samples were less than that of dusts and soil samples, their values are in an amount that can pose health risks to humans on continuous usage of the rain water, as most of the detected levels were above US EPA maximum contaminant level (MCL) of 0.015 mg/L of lead in drinking water [35]. In Nigeria, due to inadequate or non availability of potable water supply, a great number of Nigerians in both urban and rural areas depend on rain water supply for their domestic needs. The values of lead (Pb) in the early and late rain water samples in most cities were higher than those of mid-rain (Tables 3 and 4). This, agrees with weather conditions, during early and late rains (April–June and September–October), the rain samples contains dust as the atmosphere is less saturated but during mid-rain (peak of rainy season), there is high level dilution; an exception in our results is the values of Aba and Port Harcourt with near consistent values for the three measurements for the years under study. The non detection of lead in rain samples from Enugu and Awka, may depend on the volume of rainfall on the day of sampling, heavy downpour may lead to high dilution and non detection of the metal while scanty rainfall may result in significant concentration. The lead levels of dust particles were higher in Enugu, Awka, Nnewi Aba and Warri in 2008, while Onitsha and Port-Harcourt has high levels in 2007 (Figure 1), this agrees with other results reported elsewhere in Nigeria [16].

Widespread contamination of the environment with lead is consistent with the results of epidemiological studies which have found elevated blood lead levels in a large proportion of Nigerian children. In a recent study, it was shown that one quarter of the children tested in Nigeria had blood lead levels (BLLs) over 10 μg/dL and the value for about 4% of the children exceeded 20 μg/dL [17]. Paint chips, soil, water, infant formulae, canned and non-canned beverages [18] and pediatric syrup [36], are chief exposure routes for lead poisoning in children. A number of non-traditional sources of lead which have been associated with elevated blood lead levels (BLLs) in Nigerian children include use of lead-containing eye cosmetics [37] and herbal medicaments [19]. Imported glazed ceramics (drinking mugs, soup bowls, and cooking pots) in Nigeria has been reported to contain more lead than those that are locally manufactured [38]. Major adult exposure includes food, which is believed to account for over 60% of blood levels; air inhalation accounts for nearly 30% while water is of 10% [39]. We have previously shown that petrol based occupations are a major exposure pathway in Nigeria, urine samples of occupationally exposed artisans (auto-mechanics, automobile-“Okada” riders, fuel attendants in filling stations) and students as control [11].

Environmental exposure to lead poses risks of intellectual impairment, poor educational attainment, and lowered lifetime achievement [40–42] for current and future generations of children in the country. Lead poisoning is also capable of inhibiting the final step in heme synthesis by interfering with the activity of the enzymes δ-aminolevulinic dehydratase (ALAD) and ferrochelatase leading to zpp accumulation [43–45]. All these and the long history of lead poisoning provide many lessons about the process by which scientific knowledge is translated into public health policy. We suggest that the African public health community strengthen their efforts to prevent lead poisoning in African children through a holistic approach that includes the promulgation and enforcement of appropriate legislation as well as research on mitigation measures.

## Figures and Tables

**Table 1.** Lead levels (mg/kg) of roadside surface soil in the cities of study in 2007.

**Town**	**Road One**		**Road Two**	
	**Point One**	**Point Two**	**Point One**	**Point Two**
Enugu	10. 29 ± 0.01 (0.88)	28.20 ± 0.00 (1.64)	27.12 ± 0.01 (1.20)	17.06 ± 0.01 (0.92)
Awka	6.47 ± 0.02 (0.44)	6.31 ± 0.02 (0.21)	14.11 ± 0.00 (0.48)	14.16 ± 0.11 (0.78)
Nnewi	33.40 ± 0.01 (1.66)	13.92 ± 0.00 (0.75)	24.35 ± 0.00 (0.66)	10.29 ± 0.00 (0.28)
Aba	16.64 ± 0.25 (0.78)	17.77 ± 0.07 (1.32)	22.56 ± 0.01 (2.12)	17.36 ± 0.02 (1.02)
Onitsha	120.00 ± 0.00 (4.22)	38.99 ± 0.01 (2.10)	19.60 ± 0.00 (0.87)	14.78 ± 0.00 (0.48)
Port-Harcourt	7.43 ± 0.00 (0.24)	12.82 ± 0.00 (0.36)	13.08 ± 0.01 (0.56)	9.23 ± 0.01 (0.38)
Warri	8.00 ± 0.00 (0.08)	4.75 ± 0.00 (0.02)	4.75 ± 0.00 (0.04)	8.75 ± 0.00 (0.06)

N = 3, Control values in parenthesis.

**Table 2.** Lead levels (mg/kg) of roadside surface soil in the cities of study in 2008.

**Town**	**Road One**		**Road Two**	
	**Point One**	**Point Two**	**Point One**	**Point Two**
Enugu	14.50 ± 0.01 (2.85)	12.06 ± 0.01 (1.06)	10.00 ± 0.01 (0.78)	11.96 ± 0.01 (1.12)
Awka	18.82 ± 0.00 (1.96)	18.14 ± 0.01 (0.76)	16.47 ± 0.02 (1.89)	15.96 ± 0.01 (0.64)
Nnewi	44.09 ± 0.00 (1.12)	82.28 ± 0.01 (3.21)	119.20 ± 0.00 (4.67)	4,238.29 ± 0.00 (8.44)
Aba	16.41 ± 0.01 (0.98)	13.08 ± 0.01 (1.53)	21.28 ± 0.00 (0.44)	17.18 ± 0.01 (1.32)
Onitsha	16.13 ± 0.01 (1.32)	10.96 ± 0.00 (0.42)	10.97 ± 0.01 (0.22)	80.36 ± 0.00 (1.42)
Port-Harcourt	30.51 ± 0.02 (5.87)	21.28 ± 0.01 (0.36)	89.75 ± 0.03 (1.32)	12.31 ± 0.01 (0.12)
Warri	7.75 ± 0.67 (0.82)	5.00 ± 0.00 (0.12)	8.75 ± 0.00 (0.24)	6.00 ± 0.00 (0.07)

N = 3, Control values in parenthesis.

**Table 3.** Lead levels (mg/L) of rainwater in the cities of study in 2008.

**S/NO**	**Towns**	**Early Rain (April-June)**	**Mid Rain (July-August)**	**Late Rain (September-October)**
1	Enugu	Nd	Nd	Nd
2	Awka	Nd	Nd	Nd
3	Onitsha	0.053 ± 0.036	Nd	0.043 ± 0.000
4	Nnewi	0.020 ± 0.000	Nd	0.057 ± 0.000
5	Aba	0.021 ± 0.014	0.050 ± 0.000	0.027 ± 0.000
6	Port-Harcourt	0.070 ± 0.000	0.020 ± 0.000	0.036 ± 0.014
7	Warri	0.030 ± 0.000	0.010 ± 0.000	0.030 ± 0.001

N = 3, Nd = Not detected.

**Table 4.** Lead levels (mg/l) of rainwater in the cities of study in 2007.

**S/NO**	**Towns**	**Early Rain (April–June)**	**Mid Rain (July–August)**	**Late Rain (September–October)**
1	Enugu	Nd	Nd	Nd
2	Awka	Nd	Nd	Nd
3	Onitsha	0.02 ± 0.001	Nd	0.103 ± 0.000
4	Nnewi	0.033 ± 0.000	Nd	0.020 ± 0.013
5	Aba	0.023 ± 0.012	0.017 ± 0.001	0.163 ± 0.046
6	Port-Harcourt	0.077 ± 0.050	0.007 ± 0.001	0.050 ± 0.000
7	Warri	0.026 ± 0.001	0.010 ± 0.013	0.037 ± 0.000

N = 3, Nd = Not detected.

**Table 5.** The value of CEC of the soil in CM_O_l_C_/kg for the cities in 2007 and 2008.

	**2007**				**2008**			
**Town**	**Road One**		**Road Two**		**Road Three**		**Road Four**	
	**Point One**	**Point Two**	**Point One**	**Point Two**	**Point One**	**Point Two**	**Point One**	**Point Two**
Enugu	1.86 (0.36)	1.42 (0.41)	1.28 (0.34)	0.98 (0.12)	0.88 (0.13)	1.20 (0.35)	1.02 (0.72)	0.89 (0.12)
Awka	2.02 (0.54)	1.46 (0.84)	1.12 (0.21)	1.21 (0.62)	1.32 (0.24)	1.28 (0.62)	1.12 (0.62)	1.06 (0.84)
Nnewi	3.06 (0.96)	2.08 (0.22)	1.38 (0.18)	2.24 (0.64)	2.08 (0.52)	2.02 (0.71)	1.76 (0.68)	1.42 (0.21)
Aba	1.08 (0.16)	2.01 (0.18)	1.06 (0.28)	0.98 (0.28)	1.02 (0.30)	0.98 (0.28)	0.88 (0.24)	1.04 (0.46)
Onitsha	2.40 (0.54)	1.75 (0.18)	1.32 (0.28)	1.08 (0.34)	1.26 (0.38)	1.06 (0.32)	1.03 (0.41)	1.12 (0.84)
Port Harcourt	1.14 (0.10)	0.98 (0.26)	0.84 (0.19)	0.84 (0.28)	0.96 (0.32)	1.02 (0.54)	0.83 (0.21)	0.92 (0.14)
Warri	1.03 (0.43)	0.76 (0.05)	0.65 (0.09)	0.79 (0.11)	0.82 (0.14)	0.88 (0.07)	0.64 (0.08)	0.56 (0.11)

Control values in parenthesis.

**Table 6.** Showing the pH of the soil in the cities under study for both 2007 and 2008.

	**2007**				**2008**			
**Town**	**Road One**		**Road Two**		**Road Three**		**Road Four**	
	**Point One**	**Point Two**	**Point One**	**Point Two**	**Point One**	**Point Two**	**Point One**	**Point Two**
Enugu	6.20 (5.90)	6.30 (6.28)	6.60 (6.65)	6.48 (6.50)	6.30 (6.25)	6.68 (6.70)	6.60 (6.82)	6.58 (6.46)
Awka	6.60 (6.50)	6.58 (6.60)	6.61 (6.40)	6.40 (6.28)	6.62 (6.58)	6.10 (6.10)	6.50 (6.47)	6.56 (6.48)
Nnewi	6.50 (6.46)	7.20 (6.80)	6.90 (7.10)	7.18 (7.10)	6.70 (6.68)	6.30 (6.20)	6.20 (6.14)	6.38 (6.28)
Aba	7.10 (6.58)	7.40 (6.90)	6.18 (6.20)	7.10 (6.90)	7.14 (6.86)	6.20 (6.30)	6.92 (6.76)	6.70 6.68
Onitsha	7.20 (7.14)	6.80 (6.78)	6.14 (6.20)	6.12 (6.59)	6.50 (6.46)	6.80 (6.50)	6.68 (6.70)	7.50 (6.90)
Port Harcourt	6.60 (6.50)	6.50 (6.48)	6.45 (6.50)	7.10 (6.98)	7.30 (7.20)	6.14 (6.20)	6.70 (6.96)	7.56 (7.30)
Warri	6.30 (6.28)	6.20 (6.30)	6.12 (6.18)	6.20 (6.20)	6.90 (6.80)	6.58 (6.46)	6.60 (6.50)	6.80 (6.76)

Control values in parenthesis.

**Table 7.** Traffic volumes in the cities of study in 2007 and 2008 taken from 7.00 am to 6.00 pm.

	**2007**		**2008**	

**Town**	**Road One**	**Road Two**	**Road One**	**Road Two**

Enugu	(2,054)*	(1,876)*	(1,980)*	(2,060)*
	(1,800)^o^	(1,756)^o^	(2,000)^o^	(1,900)^o^
	(204)^xx^	(305)^xx^	(199)^xx^	(186)^xx^
	(306)^oo^	(286)^oo^	(276)^oo^	(204)^oo^
	(3,056)**	(2,946)**	(3,000)**	(2,908)**
	**7,420**	**7,169**	**7,455**	**7,258**

Awka	(2,000)*	(2,064)*	(2,006)*	(2,064)*
	(1,700)^o^	(1,650)^o^	(1658)^o^	(1708)^o^
	(200)^xx^	(207)^xx^	(209)^xx^	(301)^xx^
	(286)^oo^	(300)^oo^	(320)^oo^	(326)^oo^
	(2,904)**	(2,870)**	(2,680)**	(2,700)**
	**7,090**	**7,091**	**6,873**	**7,099**

Nnewi	(3,006)*	(2,986)*	(2,870)*	(2,970)*
	(2,066)^o^	(2,126)^o^	(2,240)^o^	(2,040)^o^
	(308)^xx^	(288)^xx^	(306)^xx^	(305)^xx^
	(466)^oo^	(402)^oo^	(388)^oo^	(378)^oo^
	(5,061)**	(5,461)**	(4,870)**	(4,900)**
	**10,907**	**11,263**	**10,674**	**10,593**

Aba	(2,846)*	(3,056)*	(2,800)*	(2,780)*
	(2,206)^o^	(2,380)^o^	(1,986)^o^	(2020)^o^
	(320)^xx^	(414)^xx^	(300)^xx^	(305)^xx^
	(566)^oo^	(496)^oo^	(457)^oo^	(450)^oo^
	(5,746)**	(5,328)**	(5,208)**	(5,250)**
	**11,684**	**11,774**	**10,751**	**10,805**

Onitsha	(1,800)*	(1,856)*	(1,780)*	(1,920)*
	(1,700)^o^	(1,690)^o^	(1,908)^o^	(1,780)^o^
	(208)^xx^	(205)^xx^	(200)^xx^	(220)^xx^
	(600)^oo^	(538)^oo^	(493)^oo^	(420)^oo^
	(5,605)**	(4,780)**	(4,860)**	(5,010)**
	**9,913**	**9,069**	**9,241**	**9,350**

Port Harcourt	(3,000)*	(3,004)*	(3,200)*	(2,062)*
	(1,600)^o^	(1750)^o^	(1,700)^o^	(1,720)^o^
	(300)^xx^	(306)^xx^	(250)^xx^	(252)^xx^
	(405)^oo^	(402)^oo^	(387)^oo^	(356)^oo^
	(4,085)**	(4,026)**	(4,000)**	(4,200)**
	**9,390**	**9,488**	**9,537**	**8,590**

Warri	(2,846)*	(2,700)*	(2,600)*	(2,650)*
	(1956)^o^	(1,850)^o^	(1,820)^o^	(1,780)^o^
	(285)^xx^	(486)^xx^	(280)^xx^	(276)^xx^
	(564)^oo^	(276)^oo^	(426)^oo^	(410)^oo^
	(4,846)**	(5,020)**	(5,000)**	(5,200)**
	**10,4 97**	**10,332**	**10,106**	**10,316**

( )* = Private cars,

( )^o^ = Taxis,

( )^xx^ = Mini buses,

( )^oo^ = Trucks,

( )** =Motor cycles, Bold values are the total figures.
